# A PLS-Neural Network Analysis of Motivational Orientations Leading to Facebook Engagement and the Moderating Roles of Flow and Age

**DOI:** 10.3389/fpsyg.2020.01869

**Published:** 2020-08-07

**Authors:** Inma Rodríguez-Ardura, Antoni Meseguer-Artola

**Affiliations:** Department of Economics and Business, Open University of Catalonia, Barcelona, Spain

**Keywords:** engagement, Facebook, enjoyment, self-presentation, community belonging, flow, age

## Abstract

Despite engagement being a criterion for the success of initiatives on Facebook, there is a lack of conclusive evidence about its connections with the psychological and motivational orientations that lead one to use Facebook. Built upon the uses and gratifications theory, we develop an integrative and context-specific model that links engagement with enjoyment, self-presentation, and community belonging−identified as motivational orientations underlying Facebookers’ behaviors. We also draw on current flow accounts and socioemotional selectivity theory to examine the potential moderating roles of both flow experiences and age differences. We validate the survey instrument and test the model on a sample of active Facebook users. Model testing and sensitive analysis is performed with a two-stage method that combines partial least squares (PLS) and artificial neural network analysis. The results provide strong support for the validity of the hypothesized causal, mediating and moderating relationships embodied in the model. The research also provides insights into practitioners seeking to enhance Facebookers’ engagements and promote continued use of Facebook.

## Introduction

Facebook is the world’s biggest social networking service (SNS), both in terms of active users per month and geographical reach. Although there is the systemic and ongoing issue of misinformation, Facebook continues to reach high diffusion rates in the United States and European Union markets (Isaac, 2018), with skyrocketing growth in other regions around the world. As of March 31, 2020, Facebook had 2.6 billion monthly active users worldwide, which represents an increase of 105 million users from the previous quarter (Facebook, 2020).

For organizations and brands, engaging a large number of users through a brand’s content and services on Facebook is considered to be one of their integrated marketing communication programs’ most critical success factors (Boyd and Forbes Agency Council, 2018). Engagement manifests itself as positive affective feelings and motivationally directed behaviors (Hollebeek et al., 2016) through which an individual volitionally connects and contributes, directly or indirectly, to a community, brand, or organization (Pansari and Kumar, 2017). On Facebook, a user’s engagement behaviors go beyond commercial exchanges and might consist of practices such as: providing creative feedback and assistance to other users in their personal social network; producing imaginative stories or content about a common interest, an event or a brand with whom the user identifies; fostering communication and interaction amongst fellow Facebookers; and willingly replying to calls, stories, or posts by other users.

From both a practical and theoretical point of view, understanding the motivational orientations that lead people to engage in Facebook is of great importance (Verhagen et al., 2015). Users’ motivations give them reason and purpose to interact on Facebook. Many studies have primarily connected users’ social participation on Facebook to two psychological motivational drivers: people’s need for self-presentation and their need to belong (Seidman, 2013; van Dijck, 2013). Recent studies have highlighted a third pervasive motivational element that makes Facebook an appealing SNS for its users: the need for entertainment and intrinsic enjoyment (Rodríguez-Ardura and Meseguer-Artola, 2018).

However, very little is known about how these central motivational drivers contribute to engagement formation (Verhagen et al., 2015). Even though the concept of engagement has increasingly drawn scholarly attention in recent years (Pansari and Kumar, 2017), previous research’s focus has been on the conceptual delimitation and measurement of engagement (Hollebeek et al., 2014; Baldus et al., 2015), taxonomies of engagement practices (Hollebeek et al., 2017; Eigenraam et al., 2018), the fit of the engagement concept with service-dominant logic and value co-creation frameworks (Jaakkola and Alexander, 2014; Hollebeek et al., 2016), and the contribution of engagement to business performance (Harmeling et al., 2017; Huang and Chen, 2018). Moreover, the handful of previous studies aimed at connecting the dots between motivation and engagement focus on a particular engagement object, such as a brand or a social venture (Hall-Phillips et al., 2016), as well as a specific context: brands’ Facebook pages (Zheng et al., 2015; Huang and Chen, 2018). Overall, this highlights the importance of examining the motivational drivers of Facebook engagement for both commercial and non-commercial content, and of doing so by adopting an integrated approach, which will be robust to small changes within, and additions to, the Facebook landscape−such as the emergence of new features, brand page functionalities, or embedded games. In this paper, we adopt this perspective, and, through the uses and gratifications theory, our first goal is to examine the interplay of motivational pillars (i.e., enjoyment, self-presentation, and community belonging) with Facebook engagement.

A couple of previous studies have focused on flow experiences’ contribution to engagement (Shin, 2018; Rodríguez-Ardura and Meseguer-Artola, 2019) but without adding motivations to the equation. Similarly, no previous study appears to have explained age’s moderating effect on the multidimensional facets of engagement that lead one to patronize Facebook; and empirical tests in media usage have offered little and sometimes contradictory evidence about the way younger and older individuals create and process SNS content (e.g., Hayes et al., 2015; Manzi et al., 2018). However, on the basis of flow accounts and socioemotional selectivity theory, it is plausible to expect that some key psychological factors’ contribution to enduring Facebook usage interacts with flow experiences and age differences. Accordingly, the second goal of this study is to determine the moderating effects of flow experiences and age.

In the present study, we develop an integrative model, new in the literature, about the dynamics of Facebook engagement. The originality of the model is threefold. First, it considers the cognitive, affective, and behavioral dimensions of engagement, and it shows, for the first time, how enjoyment, self-presentation, and community-belonging motivations trigger engagement. Second, it illustrates that flow moderates the effect of the enjoyment motivation on engagement and that age differences intensify the effect of engagement on the level of stickiness Facebook offers the individual. Third, we use a combined two-step partial least square (PLS)-neural network method to empirically test the model. This has allowed us not only to provide evidence about the causal, moderating, and mediating linear relationships triggering Facebook engagement and continuance, but also to test complex and non-linear relationships in the model.

## Theoretical Background

### User Engagement

The emergence of service-dominant logic (Vargo and Lusch, 2016), which suggests that consumers interact with media, organizations, and brands to co-create value, has come accompanied by an integrated perspective of online media consumption. This perspective conceives engagement as central to the interplay between the individuals and the medium’s, organization’s or brand’s value proposition online (Hollebeek et al., 2016). The concept of engagement is fundamental to the notion that SNS users are active participants and, sometimes, creative producers of mediated content (Lüders, 2008). This breaks with the traditional view of users as exogenous to the media, as passive recipients of mediated content and services (Bijmolt et al., 2010).

In the literature, a myriad of definitions and conceptualizations of engagement has been put forward, which overall presents engagement as a complex, multifaceted phenomenon. Some conceptualizations focus primarily on the individual’s behavior, suggesting that engagement is a conative manifestation of the individual’s satisfaction and his or her emotional closeness with a value proposition online (e.g., Junco, 2012; Jaakkola and Alexander, 2014). Under this view, engagement goes beyond a mere utilization decision and passive consumption (Harmeling et al., 2017) and translates into the individual’s contribution to the medium’s, organization’s or brand’s value proposition (Vivek et al., 2012). In contrast to this, there is a perspective that defends the notion of engagement as an internal drive that underlies an individual’s communications and collaboration activities (Baldus et al., 2015; Rodríguez-Ardura and Meseguer-Artola, 2019). This inner drive is triggered by the time and effort that the user invests in interacting with the value proposition, and it might be so exciting that it can compel him or her to display affective and behavioral reactions (Brodie et al., 2013; Zheng et al., 2015).

Added to this, a more integrated view has expanded the conceptualization of engagement and depicts it as a multidimensional, psychological mechanism (Brodie et al., 2011) that is built upon the user’s interactive experiences and which embodies cognitive, affective, and behavioral facets (Harrigan et al., 2018; Ferreira et al., 2020). Since in this paper we aim to offer a more comprehensive understanding of engagement, we adopt this latter perspective and conceive engagement as a multidimensional construct, with cognitive (i.e., knowledge-related involvement), emotional (i.e., positive affective feelings), and conative (e.g., participation, socialization) core components that result from the interaction between the individual and a value proposition online. Furthermore, we regard engagement as a volitional, desired construct (Bowden et al., 2017)−insofar as individuals voluntarily choose to devote internal resources to interact with the value proposition−and as being conceptually connected to, but distinct from, other key psychological mechanisms online (Hollebeek et al., 2016; Rodríguez-Ardura and Meseguer-Artola, 2019), such as the immersive experience of flow.

Because engagement is brought about by the user’s interactive experience with the value proposition, some confusion may arise between the concept of engagement and that of user experience. However, the experiences that users might have consist of subjective, highly immersive episodes that are not necessarily triggered by a motivational state or a particular interest (Brakus et al., 2015); instead, they help to process, give meaning to, and raise inner responses to the interplay between the user and the online value proposition (Rodríguez-Ardura and Meseguer-Artola, 2019). By contrast, engagement is motivationally based (Eigenraam et al., 2018): users choose to form thoughts, express affective feelings, and adopt behaviors due to their “intrinsic worth” (Searle et al., 2014, 381). Moreover, unlike user experiences, engagement has a behavioral basis (van Doorn et al., 2010).

Consistent with our view of engagement as a subjective episode, engagement is increasingly considered as a mediating theoretical entity rather than a final outcome (Harrigan et al., 2017; Rodríguez-Ardura and Meseguer-Artola, 2019). From this perspective, engagement reflects a user’s motivations and manifests in cognitive elaborations, positive affective feelings, and participation behaviors, which ultimately might conclude in iterative, continued usage (Eigenraam et al., 2018). So the transitional path, starting with a user’s motivational forces and finishing with continued use, might comprise cognitive, affective, and behavioral engagement (Brodie et al., 2013). A number of researchers have considered the consequences of engagement, which include the criterion variables of positive behaviors such as a higher intention to engage in continued use (So et al., 2016; Harrigan et al., 2017; Huang and Chen, 2018).

### Uses and Gratifications Theory

According to our integrated notion of engagement, engaged individuals are motivationally driven; they voluntarily invest personal resources in interactions with a value proposition (Hollebeek et al., 2016). It is precisely the uses and gratifications theory, a communication approach that examines consumer decisions in media consumption, that has the potential to explain why people deliberately perform certain behaviors in terms of their individual psychological motives.

The uses and gratifications theory presumes that people are active users of specific media channels who know their psychological needs and purposively utilize these media channels to their benefit (Katz et al., 1973; McQuail, 2012). Through the lens of this perspective, people’s actions regarding a medium or content are explained on the basis of the benefits sought (Luo and Remus, 2014), so that the mechanisms that direct people’s behavior relate to the potential benefits of using such a medium or content. This reasoning is further consistent with goal-related theoretical frameworks like the goal-setting theory (Locke and Latham, 2002), which posits that an individual’s conscious goals impel his or her subsequent actions. Similarly, Malthouse et al. (2016) built a goal-based model of user-generated online content that suggested that potential benefits operate as personal goals, the influence of which is contingent upon the active thinking they prompt. Hence, we propose that personal motivations related to Facebook usage influence engagement with Facebook.

To provide a better understanding of the diversity of psychological motivations underlying media usage, the uses and gratifications theory has summarized them in four broad categories (McQuail, 1994): entertainment-related motivations, which hedonically direct people to get intrinsic pleasure or enjoyment (Luo and Remus, 2014; Li et al., 2015); identity-related motivations, which help to express personal values and strengthen one’s self-concept (Mehdizadeh, 2010; Manzi et al., 2018); social-related motivations, which facilitate interpersonal interactions, companionship, and a sense of belonging (Wu et al., 2010; Sheldon and Bryant, 2016); and learning-related motivations, which drive individuals to discover, elaborate, and build new knowledge (Malthouse et al., 2016).

The interest drawn by SNSs and other social media (Meng et al., 2017) has allowed the uses and gratifications paradigm to continue to flourish (Quan-Haase and Young, 2014; Verhagen et al., 2015). A few studies into Facebook have shown the appropriateness of this theoretical framework and its classification of motivational drivers, except in regard to learning-related motivations (Smock et al., 2011; Li et al., 2015). As suggested by Sin (2016), SNSs like Facebook are not primarily used for knowledge acquisition purposes, but the rest of the motivational forces proposed by the uses and gratifications paradigm might come into play.

This is in sync with many studies on Facebook that have mainly related the nature of a user’s participation to the need for self-presentation and the need to belong to a community (e.g., Seidman, 2013; van Dijck, 2013; Casale and Fioravanti, 2018), as well as recent studies that have highlighted a third pervasive motivational element: the need for entertainment or intrinsic enjoyment (Hung et al., 2016; Rodríguez-Ardura and Meseguer-Artola, 2018). The motivation toward self-presentation is conceived as an inner factor that leads the user to enhance their self-concept and make a good impression on others (Krasnova et al., 2010), while the motivation toward community belonging leads the user to feel attached to other people who are important to him or her and form interpersonal bonds (Baumeister and Leary, 1995). Through storytelling and narrative self-presentations, Facebookers compound and manage standardized persona displays (van Dijck, 2013); *via* affordances that facilitate connectedness (e.g., groups, *friending*, *liking*, *following, messaging*), they foster interpersonal relationships and social acceptance amongst fellow users (Nabi et al., 2013). In addition, Facebookers entertain and have fun, not only with the pure-game resources included in the platform (Lai and Yang, 2016) but also with the use of self-presentation and social interaction functionalities in a humorous, amusing, and waggish fashion (Lewin-Jones, 2015), with memes, gags, and funny or ironic videos of celebrities and politicians shared in the news feed and with bouncy dialogs and jokes about the content at hand (Lambert, 2013).

We included these three motivational pillars (need for enjoyment, need for self-presentation, and need for community belonging) in our model based on two considerations. First, the selected potential motivations correspond to the generic categories identified in uses, and gratifications studies are supported by literature on Facebookers’ behavior and have been empirically tested. This will facilitate the soundness of the motivational forces under study and give our results a broader perspective. Second, we limited the selection of motivational elements to those discussed and validated as core motivational drivers in the literature. This will allow us to ensure that the structure of our model is both comprehensive and manageable.

### Flow Under Study

Of the diverse theoretical entities that put interactivity between the user and the value proposition at the core, flow seems to be the closest psychological mechanism to what is believed to be the “quintessence” of a user’s immersive experience (Rodríguez-Ardura and Meseguer-Artola, 2016, 508). From a user standpoint, flow episodes are exceptionally enjoyable and have intrinsic hedonic motivational drivers (Csikszentmihalyi, 1975a). This is because, for a user to experience a flow episode, he or she must be deeply engrossed in the activity being performed, and the level of satisfaction generated by such an activity is so high that it turns out to be an end in and of itself (Csikszentmihalyi, 2008). Moreover, when users go through immersive episodes of flow, they are so absorbed by the activity at hand that they lose sight of their immediate physical surroundings and track of time (Fang et al., 2013; Pelet et al., 2017).

Our decision to include flow in our model is founded on the notion that flow is particularly applicable to the realm of Facebook experiences (Kaur et al., 2016; Rodríguez-Ardura and Meseguer-Artola, 2019) and on the consensus that flow leads to satisfy the need for enjoyment (Sherry, 2004; Fang et al., 2013)−so it is a relevant component of the nomological network of engagement (Pagani and Mirabello, 2011; Shin, 2018). Flow theory offers an explanation of how and why users subjectively experience a sense of intrinsically motivating enjoyment when they involve themselves in immersive online activities and spend a long time with entertaining new media content (Weber et al., 2009). We therefore maintain that flow episodes are particularly relevant for SNSs like Facebook due to its users’ interest in fulfilling intrinsic hedonic motivations (Hung et al., 2016; Rodríguez-Ardura and Meseguer-Artola, 2018), giving room to gratifications that might be described as *enjoyment* (Lin and Lu, 2011; Reinecke et al., 2014).

### The Role of Age in Continuance Intention

Previous studies have observed age-related differences in the effects of emotionally valenced experiential stimuli when making decisions about time use and activities to be involved in (for a meta-analysis see Reed et al., 2014). This age-by-valence interaction translates into a disproportionate preference among older adults to take into consideration and process positive over negative experiential information.

Socioemotional selectivity theory (Carstensen, 2006, 2018), a lifespan theory of behavioral intentions, offers an explanation of this positivity effect among older adults. The theory posits that, with age, people are increasingly aware of the finitude of their lives, so as they grow older they adopt emotion-regulation strategies that guide them to prioritize emotionally gratifying activities and relationships (Mather and Carstensen, 2005; Martins et al., 2018), including online (Chang et al., 2015). By contrast, youngsters perceive longer and more nebulous time horizons, so they do not feel compelled to set aside activities, regardless of their valence, if they might provide valuable resources in the future (Chang et al., 2015).

Because we understand engagement as a positively valenced multidimensional mechanism (Hollebeek et al., 2014, 2016), we apply tenets from socioemotional selectivity theory to explain the part age-related differences play in users’ decisions to continue spending time and cognitive efforts on Facebook.

## Research Model and Hypotheses

**FIGURE 1:**
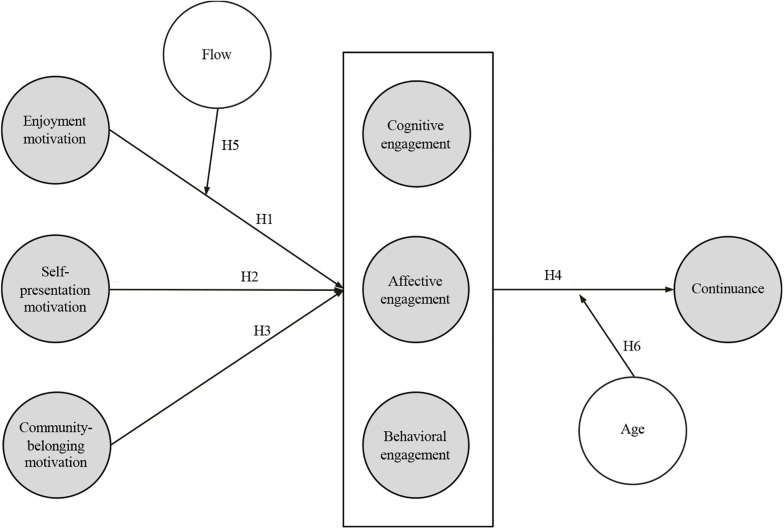
Theoretical backbone of engagement.

Figure 1 displays our conceptual model. In line with our first research goal and according to the uses and gratifications theory and existing literature on Facebook, three selected motivational forces (i.e., enjoyment, self-presentation, and community belonging) are modeled as predictors of Facebook engagement. In turn, engagement is conceived as a mediating psychological mechanism that facilitates continued use. In sync with our second research goal, and supported by accounts on flow, flow is expected to have a moderating role in engagement formation. Also, through socioemotional selectivity theory, age is considered to be a moderating variable of the engagement-continued use pathway. In the remainder of this section, we delineate the constructs in the model and justify the hypothesized relationships between them.

### Motivational Drivers Leading to Engagement

Consistent with the uses and gratifications theory (see Quan-Haase and Young, 2014), people who use Facebook on an ongoing basis are motivated−at least to some degree−by the enjoyment associated with the interactions within this SNS (Błachnio et al., 2016; Rodríguez-Ardura and Meseguer-Artola, 2018). In the case of Facebookers, enjoyment motivation refers to their drive for the playfulness, pleasure, or intrinsic fun derived from interacting online with people, games, or entertaining content (Malik et al., 2015; Hung et al., 2016).

Overall, Facebook cannot merely be characterized as a platform for social networking because it offers a wealth of entertainment (Yang and Lin, 2014), which means it has a strong potential to provide immediate enjoyment gratification (Reinecke et al., 2014; Luqman et al., 2017). These enjoyment rewards are even more likely to arise when content is produced by peer Facebookers, encourages the user’s mental imagery or sensory experiences, and appeals to user interactions (Sashittal et al., 2012; Yang and Lin, 2014), as well as when the Facebooker engrosses him- or herself in embedded games (Yang and Lin, 2014) or makes use of the co-creation or personalization functionalities at hand (Hung et al., 2016). The characteristics of the enjoyment benefits and the recreational behaviors relate to the features of engagement (Pöyry et al., 2013). Indeed, there is evidence about the contribution of perceived enjoyment to triggering engagement−either operationalized as mental involvement (Rodríguez-Ardura and Meseguer-Artola, 2018), as browsing and participation behavioral practices (Pöyry et al., 2013), or as knowledge sharing (Moghavvemi et al., 2017)−on Facebook. In light of these arguments, we propose the following hypotheses:

H1: Enjoyment motivation has a positive effect on engagement.

Self-presentation motivation−fundamental to the individual’s interpersonal, creative expression of thoughts, emotions, and experiences and the development of a sense of self (Chaudoir and Fisher, 2011)−is regarded as a major motivation for hosting a personal page on Facebook (Seidman, 2013; Błachnio et al., 2016). Self-presentation motivation has been related to the Facebookers’ interests in drawing attention and gaining a certain reputation within their personal social networks (Seidman, 2014). On Facebook, self-presentation translates into the construction of one’s own tangible identity as well as a strategic endeavor to prompt desired impressions of oneself among an intended Facebooker audience (Mimoun and Ammar, 2013; Błachnio et al., 2016).

Facebookers who aim to fulfill their needs for self-presentation are likely to convey important aspects of their selves within their digital portraits and make themselves noticeable through their Facebook contributions (van Dijck, 2013; Seidman, 2014). There is evidence suggesting that, thanks to Facebook functionalities for self-expression and self-promotion (like personal profiles crafted as narrative biographies, and status updates and posts involving cultural events, brands, causes, and trends with semiotic potential), users shape their persona portraits and establish associations with external elements that are consistent with their own self-identity (Seidman, 2013; Manzi et al., 2018). On the basis of the uses and gratifications theory, we thus argue that Facebookers who seek to have their self-presentation needs satisfied will utilize this SNS to the extent that they become engaged.

H2: Self-presentation motivation has a positive effect on engagement.

There is a consensus among many researchers, which states that the intrinsic motivation of community belonging is a core driver of Facebook use (e.g., Ha et al., 2015; Casale and Fioravanti, 2018). Sense of community belonging is driven by the human need for interpersonal attachment (Baumeister and Leary, 1995); it helps to explain relatedness functioning (Zhao et al., 2012) and might be fostered in online environments (Zhao et al., 2012; Hoffman et al., 2017). On SNSs such as Facebook, a sense of community belonging refers to the feeling of attachment, affiliation, or identification with the other members of a personal social network or group.

When Facebookers feel a certain sense of community belonging, they might be more willing to care about other people in their social network (Burke and Kraut, 2016) and interact with them (Zhao et al., 2012)−either by creating content (or communicating) about topics that matter to them, being a reliable interlocutor, or giving (or taking) emotional support (Seo et al., 2016; Liu et al., 2018). This interpersonal connectivity encourages bonding with members of the personal social network (Oh et al., 2014; Yang and Lin, 2014) and, therefore, it enhances the social and affectual reward that Facebookers perceive (Marbach et al., 2016), which in turn boosts their psychological engagement (Lee et al., 2011; Marbach et al., 2016). Accordingly, the motivational mechanism that leads one to feel emotionally close and connected to others is expected to produce relatedness gratifications and thus activate engagement.

H3: Community-belonging motivation has a positive effect on engagement.

Because we adopt an integrated approach, we conceptualize engagement as a motivated (Eigenraam et al., 2018) and positively valenced (Schamari and Schaefers, 2015; Hollebeek et al., 2016) psychological state resulting from users’ interactions (Hollebeek et al., 2016; Eigenraam et al., 2018)−often social in nature (Hollebeek et al., 2016; Eigenraam et al., 2018). However, we also give credit to the multidimensional quality of engagement, which reflects the cognitive, affective, and behavioral nature of the efforts Facebookers invest in their interactions within this SNS (Bowden et al., 2017; Harrigan et al., 2018). This multidimensional understanding of engagement gives relevance to Mollen and Wilson’s (2010), Brodie et al.’s (2013) views, looking beyond engagement as a mere state of mental involvement−or interest that facilitates the use of cognitive capabilities−and an emotional attachment to see it as encompassing actual behavioral connections within, or related to, an SNS like Facebook (Dessart et al., 2016; Harrigan et al., 2018). Accordingly, our subsequent operationalization of engagement will portray it as a superordinate construct that subsumes users’ cognitive, affective, and conative ways of becoming mentally active on, energized by, or connected with Facebook.

### Engagement Leading to Continuance

As indicated above, engagement is a psychological entity that may mediate the effects of an individual’s motivational drivers to use Facebook on his or her willingness to use this SNS long-term (Huang and Chen, 2018). In fact, loyalty to Facebook has been seen as a higher-level outcome of users’ engagement with Facebook (Huang and Chen, 2018; Rodríguez-Ardura and Meseguer-Artola, 2019).

One rationale that helps to explain the impact of engagement on continued Facebook use is social exchange theory (Homans, 1958; Blau, 2009), which holds that people become involved in social exchanges based on their perceptions of such exchanges’ worth (Homans, 1974). In line with this theoretical framework, and because engagement has a positive value for the Facebooker (Verleye et al., 2014), engagement potentially drives the individual’s decision to maximize his or her reward and repeat the interactive dynamics that created such a positive outcome (Homans, 1974), leading them, therefore, to continue their previous interactive use of Facebook. Put differently, engagement on Facebook relates to how Facebook’s value proposition is relevant for the user and thus becomes an underlying core pathway to the creation of lasting bonds with this SNS (Rodríguez-Ardura and Meseguer-Artola, 2019; Kaur et al., 2020). Based on all of the above, the position is that higher levels of engagement with Facebook will increase a Facebooker’s likelihood of continuing to use Facebook.

H4: Engagement has a positive effect on willingness to continue.

### Flow

The sense that consuming novel content, which challenges the user’s imagination, is an enjoyable and intrinsically rewarding activity is central to flow (Sherry, 2004; Baumann et al., 2016). Flow is understood to be an intrinsically enjoyable peak experience that is plunged into by users who are so deeply immersed in some particular activity online that they lose their sense of time (Csikszentmihalyi et al., 2005; Hoffman and Novak, 2009). Enjoyment, as accomplished in flow, is an “autotelic” and intrinsically rewarding experience (Asakawa, 2010), characterized by the loss of self-consciousness and a distortion of temporal orientation (Hardie-Bick and Bonner, 2016; Pelet et al., 2017). It is not surprising that an SNS like Facebook is a particularly strong enabler of the conditions that elicit flow episodes (Weber et al., 2009): a profound absorption in the events and actions happening online (Lai and Yang, 2016) and a distortion of the passage of time (Kwak et al., 2014).

Subjective experiences of flow might influence the levels of thoughts, attitudes, and conative elements related to engagement. Flow has been found to have a relevant effect on enduring involvement (McGinnis et al., 2008), participatory behavior (Pöyry et al., 2013; Chang, 2015), and further action (Kim et al., 2013; Rodríguez-Ardura and Meseguer-Artola, 2017). Seminal descriptions of flow episodes (Csikszentmihalyi, 1975b; Hoffman and Novak, 1996) show how flow is accompanied by feelings that we can relate to engagement. This is because the characteristic state of mind of a user in flow is that of an intense involvement and deep concentration on the activity they are performing online at the present moment. The activities capable of raising flow are challenging or intrinsically interesting (Rodríguez-Sánchez et al., 2011), so they require from the user a deep and focused concentration on relevant stimuli, and do not allow the individual to devote psychic energy to distractions (Rodríguez-Sánchez et al., 2011; Hamari et al., 2016). Hence, from a flow theory perspective, flow is a source for mental activation, meaningful accomplishment, and related emotions by means of stimulating activities that require high attention and engage−not only cognitively but also affectively and behaviorally (Hamari et al., 2016).

Ranaweera et al. (2005), Chang et al. (2014), Alcantara-Pilar et al. (2015) observed that, when users become wrapped up in their enjoyment of their online immersive experiences, these experiences moderate the path toward behavioral commitment. Similarly, engagement in a serious game has been reported to be moderated by the user’s experience in and of itself (Deater-Deckard et al., 2014). This is because external stimuli might alter the impact of an individual’s expectations (Chang et al., 2014). So, in the focal context of Facebook, the effect of the hedonic expectation of enjoyment might be reinforced with the actual occurrence of flow episodes. Accordingly, an intense flow experience might encourage the Facebooker to express his or her need for enjoyment as a high degree of willingness to engage. Conversely, for the user who seeks enjoyment yet finds that Facebook does not elicit flow, flow might interact negatively with this need for enjoyment.

H5: The positive effect of need for enjoyment and engagement is moderated by flow.

### Age Differences

Although psychological motivations to use Facebook do not seem to vary significantly across generations (Manzi et al., 2018), it has been found that, compared to younger Facebookers, older cohorts have less friends on Facebook (McAndrew and Jeong, 2012; Chang et al., 2015), are involved in a narrower range of Facebook activities (Mo et al., 2018) and experience less negative emotions than do younger Facebookers (Hayes et al., 2015; Settanni and Marengo, 2015). Older Facebookers are also less emotionally impacted by this SNS than their younger counterparts (Hayes et al., 2015), so they show a higher degree of emotional stability than younger Facebookers (Mo et al., 2018). Parallel to this, it has been suggested that age differences have a moderating role in a user’s decision to continue using online games (Li et al., 2015; Jang et al., 2018).

However, to the best of the authors’ knowledge, no previous study has explored whether age-related differences are a relevant element in explaining a positive association between engagement and people’s intention to continue using an SNS such as Facebook. Based on the tenets of socioemotional selectivity theory (Reed et al., 2014; Chang et al., 2015), this potential moderating role implies that, unlike younger Facebookers, older users see their life span as being shorter, so they are more inclined to choose to be involved in online activities and relationships that generate positive feelings. In other words, they might emphasize emotional well-being in the present moment and center their Facebook interactions on pleasant content, activities, and social connections that offer meaningful and immediate emotional satisfaction (Sinclair and Grieve, 2017).

This preference for positive over negative external stimuli manifests at the behavioral level (Swirsky and Spaniol, 2019)−so older Facebookers might tend to avoid negative information and look for, and choose to be exposed to, content and activities that help them to keep a positive mood. Accordingly, they will attempt to optimize engagement’s positive-valence mechanisms by implementing continuance decisions that enhance the possibility of experiencing engagement again. Therefore, we hypothesize that age strengthens the causation between engagement and continuance intention.

H6: Age has a reinforcing effect on the relationship between engagement and continuance.

## Research Methodology

### Participants

To collect the empirical data, an online survey was conducted on Facebook in Spain. Participant Facebookers were recruited *via* snowball sampling, which is a relatively high-quality method of recruitment when no list of members of the sample frame exists, and therefore it is not possible to use a probability sampling (Kosinski et al., 2015). Also, an SNS like Facebook is suited to the study of people’s experiences in this same setting (Reis and Gosling, 2010), especially when they might be related to the communication dynamics in social networks themselves (Landers and Behrend, 2015).

Participation in, and referrals to, the survey were not financially rewarded, so respondents participated out of interest and invited Facebookers with who they shared social connections. This has been related to a higher willingness to join the survey, more honest responses and yielding data of higher quality (Baltar and Brunet, 2012; Kosinski et al., 2015). In a first wave, the online questionnaires were only distributed among members of an *ad hoc* sample. However, in the following waves, participants used chain referral to promote the survey and recruit new respondents. Therefore, the sampling developed through “semi-random” recruitment (Baltar and Brunet, 2012, 70). To boost the likelihood that the subsequent waves in the snowballing process would reach diverse segments within the same sample frame (Johnston and Sabin, 2010; Morgan, 2012), the starting pool of participants was diverse.

A total of 1,285 people participated in the survey, of which 878 were removed after screening and checking for response uniqueness, questionnaire completeness, and elegibility criteria (i.e., being a monthly active Facebook user with a minimum age of 18), and also by examining the existence of insufficient effort in responding and careless response patterns (Desimone et al., 2015; Godinho et al., 2016). The size of the final sample (407) was valid for the subsequent PLS analysis since it considerably exceeded the product of 10 times the largest number of paths pointing to a particular endogenous construct in the model (Barclay et al., 1995)−which was 50 (10 × 5).

To discard non-coverage and non-response biases and verify the representativeness of the final sample, we confirmed that the differences between the demographic features of the sample and those reported for the Spanish target population were minor and non-significant. As seen in Table 1, the female/male ratio and the age structure in the sample were similar to those of the population. Furthermore, the *t*-test (in the case of gender, *p*-Value = 0.19) and the chi-squared test (in the case of the age structure, *p*-Value = 0.11) did not reveal any statistically significant differences. In addition to this, we used the multigroup comparison technique (Sarstedt et al., 2011) to check that gender did not have an interaction effect in the main model.

**TABLE 1:** Descriptive statistics of the sample.

Variables		Population (%)*	Sample (%)
Gender	Female	53.0	56.3
	Male	47.0	43.7
Age	18–39	52.0	50.3
	40–64	42.0	44.8
	≥65	6.0	4.9

*Source: The Social Media Family (2018).

### Measures

Since Facebookers’ motivations, engagement, and flow episodes reflect phenomenal experiences or psychological mechanisms that are not observable, we assessed these constructs with self-report scales (Sigerson and Cheng, 2018)−all validated by previous studies (see Table 2). To measure enjoyment motivation, we used Ghani and Deshpande’s (1994) scale. We adapted Schouten et al.’s (2007) online self-disclosure scale, partially based on Miller et al.’s (1983), to capture self-presentation motivation. A scale employed by Sánchez-Franco et al. (2015) to operationalize identification with the Facebook community was used to depict users’ community-belonging motivation. In line with Hollebeek et al. (2014), engagement was a second-order construct, and its three components were first-order factors measured by their respective indicators: the cognitive importance subscale; the affection subscale; and the community activation subscale, as developed by McQuarrie and Munson (1992); the affection subscale of the engagement scale built by Hollebeek et al. (2014); and the community Koh and Kim’s (2004) activation scale. The three items we used to measure continuance intention were originally operationalized by Moon and Kim (2001); and the other three that captured flow episodes were adapted from Novak et al. (2000). The variables were all reflective in nature and were measured with multi-item scales−using a seven-point rating scale for each item. Self-presentation motivation was measured on a scale anchored between “I do not say anything about this” and “I say everything about this”; and an item of flow (F3) was measured on a scale ranging from “never” to “always.” All remaining items were measured with a Likert-type scale ranging from “strongly disagree” to “strongly agree.”

**TABLE 2:** Measurement scales.

Constructs	Original scales	Adapted questionnaire scales
Enjoyment motivation	Ghani and Deshpande (1994)	[*Facebook is:*] (E1) Interesting (E2) Fun (E3) Exciting* (E4) Enjoyable
Self-presentation motivation	Schouten et al. (2007)	[*How much do you disclose on Facebook* (*e.g., in timeline posts*) *about?*] (SP1) Your personal feelings (SP2) The things that comfort you (SP3) Moments in your life you are proud of (SP4) Moments in your life you feel good about
Community-belonging motivation	Sánchez-Franco et al. (2015)	(CB1) I identify with my Facebook community (CB2) My opinions are valued in my Facebook community (CB3) Lots of people in my Facebook community know who I am* (CB4) I feel like my Facebook community is my own
Cognitive engagement	McQuarrie and Munson (1992)	[*I find that using Facebook:*] (CE1) Is important (CE2) Is relevant (CE3) Means a lot to me (CE4) Matters to me (CE5) Is of concern to me*
Affective engagement	Hollebeek et al. (2014)	(AE1) I feel very positive when I use Facebook (AE2) Using Facebook makes me happy (AE3) I feel good when I use Facebook (AE4) I’m proud to use Facebook
Behavioral engagement	Koh and Kim (2004)	(BE1) I take an active part in my Facebook community (BE2) I do my best to stimulate my Facebook community (BE3) I often provide information/content for my Facebook friends (BE4) I eagerly reply to posts by Facebook friends (BE5) I take care of my Facebook friends (BE6) I often respond to calls from Facebook friends who seek support
Continuance intention	Moon and Kim (2001)	(CI1) I will use Facebook on a regular basis in the future (CI2) I will frequently use Facebook in the future (CI3) I will strongly recommend others to use Facebook
Flow	Novak et al. (2000)	[*Description of flow, followed by instructions*] (F1) I have (at some time) experienced “flow” on Facebook (F2) Most of the time I use Facebook I feel that I am in flow (F3) In general, how frequently would you say you have experienced flow when you use Facebook?

*Removed after reliability analysis.

The instrument measurement was made available in the three languages most used on Facebook Spain (Owloo, 2015). The original scale items were translated through a back-translation procedure (Brislin, 1986), and pre-tested for content validity (Haynes et al., 1995). Ten scholars, all familiar with the research issue and the goals of the measurement instrument, participated in the pre-test. In addition, a pilot test was conducted with 60 university students to detect translation biases. Results of a multivariate analysis of variance showed that there were no significant differences between the constructs in the model due to the language used by the pilot participants.

### Prevention and Assessment of Common Method Variance

Validity of the survey instrument might be compromised by potential systematic method variance, which might affect item validities, reliabilities, and covariations between observed variables (MacKenzie and Podsakoff, 2012; Rodríguez-Ardura and Meseguer-Artola, 2020). This potential issue is stronger when all the constructs have been measured with self-report scales and data has been gathered from the same sample and at the same time−which is our case.

To prevent the appearance of common method bias, in the design of the measurement instrument we adopted the procedural measures suggested by Podsakoff et al. (2003). For example, we adapted the items’ wording to the focal context of Facebook (Table 2) and ensured the respondents’ anonymities and the confidentiality of their answers.

Furthermore, we applied two *post hoc* statistical techniques to discard any problematic common method variance interfering in data analysis: Harman’s single-factor test and the correlation matrix procedure. First, in the unrotated factor analysis, there is no single factor that accounts for the majority of the covariance among the measures (a minimum of three factors explains more than the 50% of the variance). Second, all the pairwise correlations between constructs are clearly below the recommended maximum value of 0.90 (Table 6).

### PLS-Neural Network Method

We assessed the predictive power of the conceptual model, along with the hypothesized relationships between constructs, using a multianalytical method. This approach integrates neural network analysis into the methodological framework of the variance-based structural equation modeling (SEM) method, known as PLS modeling (see Qin and McAvoy, 1992).

PLS is considered to be an effective, powerful technique for estimating both the relationships among the (latent) constructs in a proposed model and the connections between the constructs and the measurement scale items. Unlike the covariance-based SEM (CB-SEM) techniques, PLS neither requires big sample sizes nor that the data has a multivariate normal distribution (Reinartz et al., 2009). Furthermore, it is particularly well suited to testing complex models (Hair et al., 2017a), with higher-order latent structures and many scale items, and, combined with the product indicator approach, it is highly accurate in assessing interaction effects (Henseler and Chin, 2010). However, all SEM techniques, PLS included, presume the linearity of all causal paths (Qin and McAvoy, 1992), which might oversimplify the analysis of users’ behaviors (Leong et al., 2015).

On the other hand, the artificial intelligence method of neural network analysis is suitable for examining both linear and non-linear relationships between variables with high predictive precision (Leong et al., 2013) and does not require the data to meet key underlying assumptions of normality, homoscedasticity, linearity, and non-multicollinearity (Tan et al., 2014). Nevertheless, neural network analysis is affected by overfitting problems−that is, the network correctly recognizes existing patterns but has low accuracy with new data sets (Chong, 2013; Ahani et al., 2017)−and it does not allow causal paths between variables to be statistically assessed (Chan and Chong, 2012; Chong, 2013).

Because of the complementary advantages of PLS and neural network analysis, we combined both methods in a two-step process. First, we used the PLS framework to assess the measurement model and the statistical significance of the hypothesized (causal and moderating) relationships within the proposed structural model. Second, we extended the PLS modeling to a non-linear setting and used a neural network analysis to determine the predictive capacity of the input factors and their relative importance.

## Results

### Measurement Model

To assess the measurement model, we considered internal consistency reliability, individual item reliability, the convergent validity, and the discriminant validity. Internal consistency reliability is satisfactory (see Table 3) given that all Cronbach’s α values and Dillon-Goldstein’s ρ values are above the minimum threshold of 0.70, the first eigenvalues are all greater than 1, and all second eigenvalues are below 1.

**TABLE 3:** Internal consistency reliability.

	Num. of items	Cronbach’s α	Dillon-Goldstein’s ρ	First eigenvalue	Second eigenvalue
Enjoyment motivation	3	0.855	0.912	2.330	0.397
Self-presentation motivation	4	0.852	0.901	2.780	0.611
Community-belonging motivation	3	0.824	0.895	2.220	0.503
Cognitive engagement	4	0.887	0.922	2.990	0.472
Affective engagement	4	0.907	0.935	3.140	0.461
Behavioral engagement	6	0.891	0.917	3.900	0.884
Continuance intention	3	0.859	0.915	2.350	0.465
Flow	3	0.895	0.935	2.480	0.289

In the initial exploratory analysis, the two items that did not meet individual item reliability criteria (E3, CE5) were removed. The loadings of the remaining items on their corresponding constructs are all higher than 0.70 (Table 4), so all communalities are greater than 0.50. In most cases, the constructs retain more than 70% of the item’s variability. In addition, the convergent validity of the measures is satisfactory since the average variance extracted (AVE) of each construct is clearly above the minimum recommended 0.50 cut-off.

**TABLE 4:** Individual item reliability and convergent validity.

	AVE	Weight	Loading	Communality
Enjoyment motivation	0.775			
E1		0.371	0.867	0.751
E2		0.397	0.908	0.824
E4		0.367	0.867	0.751
Self-presentation motivation	0.694			
SP1		0.302	0.789	0.622
SP2		0.307	0.809	0.655
SP3		0.310	0.881	0.776
SP4		0.282	0.851	0.724
Community-belonging motivation	0.741			
CB1		0.405	0.896	0.803
CB2		0.405	0.885	0.784
CB4		0.350	0.797	0.635
Cognitive engagement	0.746			
CE1		0.271	0.851	0.725
CE2		0.271	0.837	0.700
CE3		0.307	0.884	0.782
CE4		0.307	0.883	0.779
Affective engagement	0.784			
AE1		0.275	0.889	0.790
AE2		0.301	0.928	0.861
AE3		0.304	0.922	0.850
AE4		0.247	0.796	0.634
Behavioral engagement	0.650			
BE1		0.223	0.843	0.710
BE2		0.202	0.799	0.638
BE3		0.197	0.807	0.651
BE4		0.208	0.842	0.708
BE5		0.217	0.820	0.672
BE6		0.193	0.721	0.519
Continuance intention	0.782			
CI1		0.382	0.916	0.839
CI2		0.370	0.902	0.813
CI3		0.380	0.832	0.693
Flow	0.826			
F1		0.328	0.888	0.788
F2		0.376	0.914	0.836
F3		0.395	0.924	0.854

To assess the discriminant validity of the measurement model, we used the cross loadings of the items and Fornell and Larcker’s (1981) criterion. First, all item loadings on their respective constructs are higher than their loadings on the rest of the constructs (Table 5). Second, the AVE square root value of each latent construct is greater than its correlations with other constructs (Table 6). Third, we evaluated the discriminant validity with the heterotrait-monotrait ratio (HTMT). As seen in Table 6, all values are clearly below the conservative threshold of 0.85 (Kline, 2011). Based on these results, we deemed that the measurement model satisfies the discriminant validity.

**TABLE 5:** Cross loadings of items.

	Enjoyment motivation	Self-pre-sentation motivation	Community-belonging motivation	Cognitive engagement	Affective engagement	Behavioral engagement	Continuance intention	Flow
E1	**0.867**	0.309	0.272	0.466	0.352	0.380	0.471	0.239
E2	**0.908**	0.348	0.373	0.465	0.413	0.402	0.494	0.303
E4	**0.867**	0.311	0.401	0.402	0.432	0.350	0.477	0.229
SP1	0.277	**0.789**	0.245	0.397	0.343	0.418	0.263	0.348
SP2	0.306	**0.809**	0.344	0.339	0.363	0.473	0.379	0.299
SP3	0.320	**0.881**	0.331	0.373	0.364	0.451	0.363	0.373
SP4	0.319	**0.851**	0.305	0.327	0.351	0.403	0.352	0.359
CB1	0.332	0.321	**0.896**	0.317	0.465	0.439	0.463	0.290
CB2	0.387	0.328	**0.885**	0.297	0.450	0.482	0.454	0.253
CB4	0.301	0.302	**0.797**	0.293	0.402	0.364	0.396	0.250
CE1	0.447	0.336	0.274	**0.851**	0.391	0.428	0.446	0.318
CE2	0.451	0.330	0.318	**0.837**	0.353	0.439	0.435	0.287
CE3	0.399	0.411	0.317	**0.884**	0.449	0.492	0.453	0.480
CE4	0.454	0.410	0.304	**0.883**	0.428	0.505	0.500	0.398
AE1	0.391	0.357	0.419	0.418	**0.889**	0.588	0.482	0.381
AE2	0.444	0.395	0.495	0.425	**0.928**	0.592	0.511	0.378
AE3	0.456	0.391	0.492	0.418	**0.922**	0.596	0.535	0.371
AE4	0.297	0.368	0.397	0.410	**0.796**	0.597	0.417	0.345
BE1	0.366	0.477	0.384	0.540	0.603	**0.843**	0.536	0.438
BE2	0.299	0.451	0.358	0.488	0.612	**0.799**	0.473	0.411
BE3	0.335	0.437	0.347	0.414	0.474	**0.807**	0.474	0.351
BE4	0.350	0.419	0.395	0.392	0.535	**0.842**	0.543	0.341
BE5	0.356	0.411	0.490	0.428	0.549	**0.820**	0.541	0.337
BE6	0.368	0.339	0.443	0.346	0.446	**0.721**	0.534	0.221
CI1	0.532	0.347	0.508	0.443	0.477	0.568	**0.916**	0.259
CI2	0.473	0.389	0.415	0.450	0.495	0.573	**0.902**	0.312
CI3	0.441	0.345	0.425	0.515	0.489	0.559	**0.832**	0.321
F1	0.224	0.338	0.287	0.357	0.313	0.371	0.300	**0.888**
F2	0.250	0.366	0.261	0.407	0.388	0.400	0.293	**0.914**
F3	0.318	0.418	0.290	0.414	0.425	0.416	0.324	**0.924**

**TABLE 6:** Discriminant validity analysis*.

	Enjoyment motivation	Self-presentation motivation	Community-belonging motivation	Cognitive engagement	Affective engagement	Behavioral engagement	Continuance intention	Flow
Enjoyment motivation	**0.880**	0.429	0.471	0.581	0.510	0.492	0.637	0.331
Self-presentation motivation	0.367	**0.833**	0.439	0.494	0.486	0.600	0.476	0.471
Community-belonging motivation	0.396	0.368	**0.861**	0.411	0.589	0.581	0.604	0.358
Cognitive engagement	0.505	0.432	0.351	**0.864**	0.525	0.605	0.608	0.480
Affective engagement	0.453	0.427	0.511	0.471	**0.885**	0.745	0.623	0.459
Behavioral engagement	0.429	0.525	0.500	0.541	0.668	**0.806**	0.733	0.485
Continuance intention	0.546	0.408	0.510	0.532	0.551	0.642	**0.884**	0.384
Flow	0.293	0.414	0.307	0.434	0.416	0.436	0.337	**0.909**

*HTMT above the diagonal, square root of the AVE on the diagonal (bold), and correlations between the dimensions under the diagonal.

### Structural Model

To measure the second-order molar construct of *engagement* we adopted the repeated indicators approach. The construct was reflectively associated with its three dimensions (i.e., *cognitive engagement, affective engagement, behavioral engagement*) by using all their items and, following Becker et al.’s (2012) recommendation, we used mode A to measure the higher-order indicator. We applied a centroid inner weighting scheme in the PLS algorithm.

We employed the product indicator approach to examine the hypothesized moderating relationships in the structural model (*H5*, *H6*). Accordingly, the interaction constructs were defined as the product between the items of the corresponding predictor and the associated moderator variable. To measure the moderation influence without possible undesired inflation effects (Hair et al., 2017b) we included in the model the moderator variables’ direct effect on the related endogenous construct (*flow* →*engagement*, *age* → *continuance*).

We validated the structural model by analyzing the coefficient of determination (*R*^2^) of the two regressions in the model, the effect size of the exogenous constructs on the endogenous constructs (*f*^2^), the standardized root mean residual (SRMR), the blindfolding-based cross-validated redundancy measure *Q*^2^ and the statistical significance of the path coefficients. The value of each coefficient of determination is between 0.25 and 0.75 (Table 7), which shows an acceptable or moderate level of predictive accuracy. In addition, the higher-order construct *engagement* is perfectly explained through its three dimensions (*R*^2^ = 1).

**TABLE 7:** Regressions with causal path coefficients.

			Estimate	Std. error	*t*-Value	*p*-Value	*f*^2^	*R*^2^	*Q*^2^
**Auxiliary regression**								1.000	
*Intercept*			0.000	*0.000*	*0.000*	*1.000*			
Cognitive engagement	→	Engagement	0.332	0.000	4790.000	0.000			
Affective engagement	→	Engagement	0.371	0.000	4710.000	0.000			
Behavioral engagement	→	Engagement	0.475	0.000	5750.000	0.000			
**Regression 1**								0.560	0.435
*Intercept*			*0.000*	*0.033*	*0.000*	*1.000*			
Flow	→	Engagement	−0.032	0.135	−0.239	0.811	0.000		
Enjoyment motivation	→	Engagement	0.147	0.069	2.120	0.035	0.268		
Flow x Enjoyment motivation	→	Engagement	0.340	0.162	2.100	0.036	0.111		
Self-presentation motivation	→	Engagement	0.254	0.039	6.560	0.000	0.105		
Community-belonging motivation	→	Engagement	0.264	0.038	6.980	0.000	0.118		
**Regression 2**								0.479	0.523
*Intercept*			*0.000*	*0.036*	*0.000*	*1.000*			
Age	→	Continuance	0.239	0.115	2.080	0.038	0.012		
Engagement	→	Continuance	0.928	0.113	8.220	0.000	0.167		
Age × Engagement	→	Continuance	−0.340	0.151	−2.250	0.025	0.012		

We examined the impact of the predictor variables on their associated endogenous constructs through their *f*^2^ effect sizes, which show that *enjoyment motivation* has the most relevant effect on *engagement* and that *engagement* is the most important predictor of *continuance*. In addition, taking Cohen’s cut-off values (1988) into consideration, we observe that *enjoyment* motivation has a medium effect on *engagement* while the rest of constructs have a low or very small effect. Similarly, *engagement*’s impact on *continuance* is medium whereas the other two predictors have a very small effect.

The Stone-Geiser’s *Q*^2^’s values indicate that the predictive relevance of the path model for the endogenous latent variable is moderate (0.44) for *engagement* and high (0.52) for *continuance* (Hair et al., 2019). The structural model’s SRMR is 0.09, which is below the recommended upper limit of 0.10 (Williams et al., 2009). Although this threshold is provisional and requires further analysis (Benitez et al., 2020), the SRMR’s value is small and indicative of the structural model’s validity.

**FIGURE 2:**
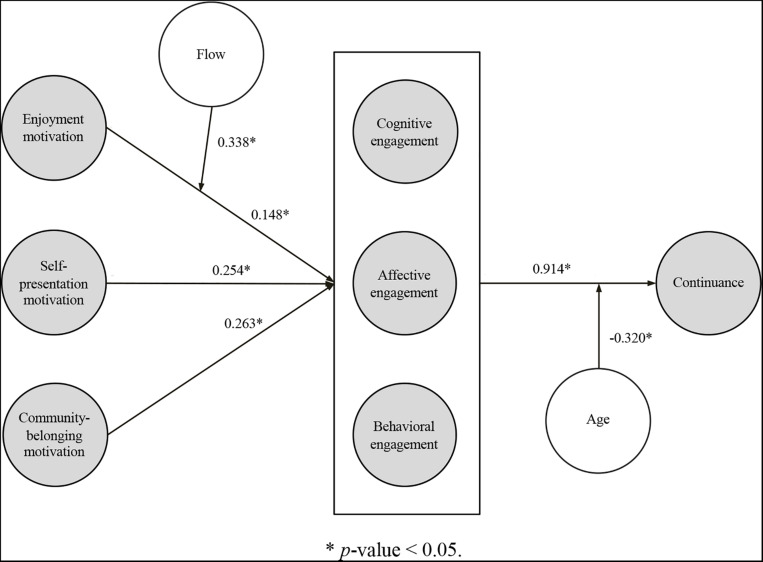
PLS model with path coefficients.

Since data does not follow a multivariate normal distribution, we used the bootstrap resampling procedure (with 500 resamples) to test the statistical significance of the path coefficients (Table 8). All *p*-Values are below 0.05 and the corresponding Benjamini-Hochberg alpha correction, except for the *p*-Values associated with the moderator variables’ direct effects on the endogenous construct. Accordingly, we can deem that all hypothesized causal and moderating links are supported (Figure 2).

**TABLE 8:** Results from bootstrap resampling procedure.

			Path coefficients (original) β	Path coefficients (boot-strapping)	Std. error	*p*-Value	*α* correction
Flow	→	Engagement	−0.032	−0.031	0.131	0.388	0.050
Enjoyment motivation	→	Engagement	0.147	0.148	0.065	0.029	0.031
Flow × Enjoyment motivation	→	Engagement	0.340	0.338	0.141	0.023	0.025
Self-presentation motivation	→	Engagement	0.254	0.254	0.041	0.000	0.019
Community-belonging motivation	→	Engagement	0.264	0.263	0.038	0.000	0.013
Age	→	Continuance	0.239	0.227	0.136	0.099	0.044
Engagement	→	Continuance	0.928	0.914	0.119	0.000	0.006
Age × Engagement	→	Continuance	−0.340	−0.320	0.146	0.036	0.038

*Enjoyment motivation, self-representation motivation*, and *community-belonging motivation* all have a significant, positive effect on *engagement* (β = 0.15, β = 0.25, and β = 0.26, respectively)−while the former causal relationship is indeed moderated by *flow* (β = 0.34). The higher-order construct *engagement* (measured as a compound perception of *cognitive engagement, affective engagement*, and *behavioral engagement*) has a direct and significant impact on continuance (β = 0.91), which in turn is moderated by age (β = –0.32).

All the indirect effects included in the proposed model are also significant (see bootstrapping results in Table 9). The three psychological motivations (*enjoyment motivation, self-representation motivation*, and *community-belonging motivation*) have an indirect effect on *continuance*. The indirect influence of enjoyment motivation is again moderated by flow.

**TABLE 9:** Indirect impacts between constructs.

Relationships			Indirect effects
Enjoyment motivation	→	Continuance	0.136
Flow × Enjoyment motivation	→	Continuance	0.315
Self-presentation motivation	→	Continuance	0.236
Community-belonging motivation	→	Continuance	0.245

### Neural Network Analysis

After using PLS to statistically test the causal and moderating relationships in the structural model, we integrated the neural network analysis with the PLS framework (Qin and McAvoy, 1992) so as to detect possible non-linear relationships and determine the importance of each factor (Ahani et al., 2017). Accordingly, we introduced the factors resulting from PLS analysis as significant and reliable inputs in the neural network; to boost performance (Negnevitsky, 2017), we adopted the min-max scale method to scale all data factors between 0 and 1.

**FIGURE 3:**
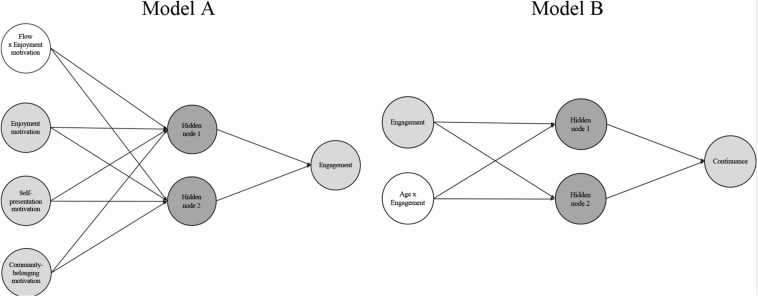
Neural network models.

Since the general structural model has two endogenous constructs (i.e., *engagement* and *continuance*), we divided it into two subneural network models: model A and model B (Figure 3). In model A, the output variable was the endogenous construct *engagement* and the input variables were: the three constructs of the general model with significant influence on *engagement* (i.e., *enjoyment motivation, self-representation motivation* and *community-belonging motivation*); and the latent variable that captures the moderating effect of *flow* on the path from *enjoyment motivation* to *engagement*. The output variable of model B was *continuance*, and its input variables were *engagement* and the latent variable that represented the moderating role of *age* in the relationship between *engagement* and *continuance*.

To each submodel, we applied a neural network multilayer perceptron training algorithm, which had a single hidden layer to represent the continuous functions of the input nodes (Hornik et al., 1989; Negnevitsky, 2017). We used the traditional backpropagation algorithm with the logistic activation function provided in *R* by the *neuralnet* package (Günther and Fritsch, 2012), and the sum of squared errors as the differentiable error function to minimize.

We established the number of nodes in the hidden layer of each submodel based on two key restrictions (Negnevitsky, 2017; Liébana-Cabanillas et al., 2018): (1) a limited number of hidden nodes does not allow complex patterns to be detected; and (2) a high number of hidden nodes can trigger overfitting issues. To satisfy both restraints, we selected the smallest number of hidden neurons possible to ensure a suitable generalization of the complex model. Accordingly, we first considered Blum’s (1992) proposition that the optimal number of nodes in the hidden layer is a value between the number of inputs and the number of outputs. Next, we followed a trial-and-error procedure (Chong and Bai, 2014; Sharma et al., 2015), which determined that, in both submodels, the best (prediction) results were obtained with two hidden nodes.

To prevent any potential bias caused by the tendency of neural network models to overfit (Geman et al., 1992; Prechelt, 1998), we performed a 10-fold cross-validation for each submodel, with a data set ratio of 90:10 for training and testing (Chan and Chong, 2012; Liébana-Cabanillas et al., 2017). We used the root-mean-square error (RMSE)−obtained from the 10 optimizations−to analyze each submodel’s accuracy. As seen in Table 10, the RMSE values for both the training data and testing data are acceptable in the two submodels. Therefore, we can safely establish that: the submodels are efficient and give high-precision predictions; the parameter estimations are reliable; and all input factors are appropriate for predicting the endogenous variables.

**TABLE 10:** Neural network prediction accuracy.

Network	Model A		Model B	
	Training	Testing	Training	Testing
1	0.122	0.129	0.162	0.169
2	0.120	0.143	0.164	0.187
3	0.122	0.129	0.166	0.137
4	0.127	0.123	0.167	0.159
5	0.128	0.122	0.164	0.157
6	0.125	0.104	0.169	0.143
7	0.124	0.106	0.164	0.149
8	0.124	0.108	0.163	0.160
9	0.122	0.140	0.165	0.149
10	0.126	0.118	0.165	0.148
Mean	0.124	0.122	0.165	0.156
s.d.	0.003	0.014	0.002	0.015

We assessed the importance of each input factor on output variability by considering the average relative importance and the normalized importance and performed this sensitivity analysis with Garson’s (1991) algorithm. To obtain the importance average, we used the results for each of the 10 networks; we calculated the normalized importance of each factor as the proportion of its relative importance with respect to the factors’ maximum relative importance (Leong et al., 2013; Sharma et al., 2015). Table 11 shows that the most important factor in predicting *engagement* is *community-belonging motivation*, followed by the moderating effect *flow x enjoyment motivation*, and also that the most important factor in predicting *continuance* is *engagement*.

**TABLE 11:** Neural network sensitivity analysis.

Network	Model A				Model B	
	Enjoyment motivation	Flow × Enjoyment motivation	Self-pre-sentation motivation	Community-belonging motivation	Engagement	Age × Engagement
1	0.172	0.252	0.187	0.389	0.712	0.288
2	0.197	0.237	0.184	0.383	0.882	0.118
3	0.148	0.288	0.208	0.356	0.710	0.290
4	0.274	0.265	0.261	0.199	0.859	0.141
5	0.157	0.466	0.199	0.178	0.700	0.300
6	0.182	0.257	0.189	0.373	0.959	0.041
7	0.192	0.259	0.179	0.370	0.724	0.276
8	0.200	0.217	0.215	0.368	0.716	0.284
9	0.112	0.196	0.206	0.486	0.734	0.266
10	0.101	0.204	0.145	0.550	0.719	0.281
Average importance	0.173	0.264	0.197	0.365	0.772	0.228
Normalized importance (%)	47.397	72.328	53.972	100.000	100.000	29.534

## Discussion and Conclusion

The main purpose of this paper has been to predict Facebook engagement formation and explain the mediating effect of engagement on continuance use. On the grounds of the uses and gratifications theory, engagement literature, and social exchange theory, we hypothesized a causal path from enjoyment motivation, self-presentation motivation, and community-belonging motivation to continued Facebook use that is mediated by engagement−operationalized as a multidimensional construct. Furthermore, based on flow theory and socioemotional selectivity theory, we respectively projected that flow would interact with enjoyment motivation to trigger engagement, and age would moderate the influence of engagement on continued use. Our empirical research combined two techniques: a PLS approach that allowed us to validate the survey instrument and confirmed all the linear (direct, mediating, and moderating) hypothesized relationships; and a neural network analysis, which quantified engagement’s and continuance behavior’s sensitivity to each input factor and determined that the predictive model is highly accurate.

### Theoretical Contributions

This research makes its main contributions in four areas. First, it theoretically integrates the research stream of engagement with the uses and gratifications paradigm and tests the suitability of this combined approach in terms of its explanatory power. In recent years, researchers have applied the uses and gratifications approach to SNSs like Facebook to examine motivations’ effect on either usage or continued usage−usually measured in terms of time spent on Facebook. This previous research does not detail how the expected gratifications of using Facebook contribute to the psychological phenomena of engagement. The very few studies attempting to explain engagement, such as Verhagen et al. (2015), did not consider the cognitive, attitudinal, and behavioral nature of the construct and its mediating role in use decisions.

Our resulting research framework (motivation-engagement-continued use) extends the uses and gratifications paradigm model, not only by conceiving engagement as a multidimensional psychological phenomenon−influenced by motivational drivers−but also by offering evidence about engagement’s role as a mediating psychological mechanism in continued-use decisions. Furthermore, by conjointly assessing the various forms of engagement that Facebookers experience and why they engage, we have been able to offer a more comprehensive understanding of the psychological context of Facebookers’ interactions.

A second contribution of this paper concerns the role attributed to immersive flow experiences in the nomological network of engagement. While previous research on flow has been mainly devoted to examining antecedents and consequences of flow, the two only empirical studies about flow’s potential connection with engagement simply considered a causal path (Shin, 2018; Rodríguez-Ardura and Meseguer-Artola, 2019), and do so with engagement operationalizations restricted to measures of cognitive involvement. These prior findings were consistent with flow’s conceptual connection with the cognitive dimension of engagement (Hollebeek, 2011; Medhurst and Albrecht, 2016) and gave proof that flow and engagement are distinct phenomena. However, they did not provide evidence of how a transient psychological mechanism like flow might intensify the more enduring emotional and behavioral facets of engagement. Our findings complement this view and show that, beyond it being characterized as a temporary yet highly immersive episode, flow also acts as a psychological amplifier that facilitates engagement’s cognitive as well as affective and behavioral dimensions. Since flow experiences are enjoyable and therefore positive, they have an indirect and positive impact on the individual’s emotional engagement. In addition, because flow is characterized by a high concentration on the online activities at hand, it becomes a highly functional episode that strengthens the subjective mechanisms leading toward behavioral engagement. As a consequence, the Facebookers whose enjoyment needs are met *via* flow tend to retain greater motivation-affect-behavior consistency.

Third, this study offers a novel line of evidence regarding age-related differences across Facebookers and shows, for the first time in the literature, their moderating role in engagement’s relationship with users’ continuance decisions. This indeed indicates the applicability of socioemotional selectivity theory in SNS contexts and extends this theory’s reach to account for individual’s decisions regarding continued use. Thus, socioemotional selectivity theory complements the perspective offered by the research stream on engagement to theoretically predict age-related changes in the effects of users’ engagement.

Lastly, this is one of the few papers using a hybrid, two-stage technique that integrates PLS and neural network analysis. In contrast to CB-SEM, PLS works well under multivariate non-normal conditions and is better-suited to modeling higher-order latent structures and assessing both direct (and mediating) paths and moderating effects. However, neither PLS nor CB-SEM can detect non-linear relationships and achieve the high predictive performance levels offered by neural networks. In turn, neural networks by themselves cannot test causal relationships. The hybrid technique we used allowed us to overcome these disadvantages by embedding neural networks in the PLS framework, thus offering a highly accurate assessment of the relative (linear and non-linear) effects of each construct. Future research might employ this approach as a refined, powerful tool to assess complex structural models in online consumer behavior.

### Managerial Implications

IT practitioners and digital marketers can benefit from this research in three main ways. First, our empirical examination−adaptable to any type of content or moderated changes in the Facebook environment−highlights that, while people interact long-term on Facebook as a means of presenting themselves and connecting with others, they also use Facebook for the sake of the enjoyment derived from the interaction experiences. As a result, continued Facebook use involves a contrast that encompasses expressing and communicating with other Facebookers while interacting with the content in and of itself. Therefore, for managers and specialists building content marketing strategies, a dual focus on social and entertainment values is recommended.

Second, our results enable managers and practitioners to be aware of the importance, for the brand and the firm, of Facebookers’ engaging experiences, allowing them to make more informed decisions when implementing strategies to boost Facebookers’ engagement. Our findings hold relevance for practitioners looking to promote engagement not only as a means by which people can achieve perceived benefits−gained from interaction experiences on Facebook−but also as a precursor to continued usage. In the professional literature, engagement has typically been depicted as either a user’s repertory of digital practices (e.g., contributing activities, linking Facebook brand pages, etc.) or a user’s involving mechanism or willingness to relate. The validated integrative measurement scale used in this study provides practitioners with a tool to enhance their understanding of the multidimensional nature of Facebook engagement and indicates that there are three major ways in which people engage in Facebook (i.e., by virtue of cognitive activation, emotional activation, and activities).

Third, managers and practitioners need to be aware that Facebook engagement fits different types of users. Our study highlights this fact through the example of flow episodes and age, although brands might use their own psychographic and behavioral segmentation criteria to detect what leads each segment to achieve engagement and what facets of engagement are most relevant for them. For example, a brand targeting both older and younger users will have to be aware that their engagement strategies will more strongly affect their older users. Additionally, by running the scale of engagement used here amongst the relevant segments of a brand’s users, practitioners will be able to detect what motivational forces and engagement facets are most relevant for them. Ultimately, this will help them tailor digital marketing strategies to match each segment.

### Limitations and Future Research

Although we endeavored to maximize the quality of our work with appropriate testing procedures and validation techniques, and the unlikely prospect that spurious correlations could occur due to common method bias, our results must be interpreted taking certain research limitations into account. For example, due to time and financial constraints, our research design was cross-sectional in nature, participants were recruited with a snowball sampling strategy, and the sampling frame was constricted to a single-country−which has a particular degree of individualism/collectivism, values conventionally linked to feminine roles, and specific individual and organizational cultural dynamics (Hofstede, 1983; Spector et al., 2001). All of this limited the predictive generalization of our findings. Because the engagement ecosystem in SNSs facilitates the detailed recording of engagement activities, future empirical enquiries could seize the opportunities offered by a mixed approach that supplements survey data with big data sources. Furthermore, future research could expand on this study by considering whether new phenomena, or extensive and profound transformations in the Facebook landscape, shape users’ engagement mechanisms in the long term. Further insight could be gained by empirically testing if cultures (e.g., national cultures, consumption cultures) play a part in engagement formation. Previous cross-cultural studies have shown that, while motivational drivers to use an SNS like Facebook might be relatively similar across cultural contexts (Kim et al., 2011; Manzi et al., 2018), the way in which SNSs are used might significantly vary among cultural milieus. However, the simultaneous interplay between multiple cultural differences, psychological motivations, and behavior on Facebook remains unclear.

## Data Availability Statement

The datasets presented in this article are not readily available because participants did not give explicit consent for data sharing. Requests to access the datasets should be directed to irodriguez@uoc.edu

## Ethics Statement

The studies involving human participants were reviewed and approved by the Universitat Oberta de Catalunya’s Research Ethics Board. The participants provided their informed consent to participate in this study.

## Author Contributions

IR-A contributed to the conception and design of the study, and wrote the (sub-)sections “Introduction,” “Theoretical Background,” “Research Model and Hypotheses,” “Participants,” “Measures,” “Prevention and Assessment of Common Method Variance,” and “Discussion and Conclusion” of the manuscript. AM-A organized the database, performed the statistical analysis, and wrote the sections “Participants,” “Prevention and Assessment of Common Method Variance,” “PLS-Neural Network Method,” and “Results” of the manuscript. Both authors contributed to manuscript revision, read, and approved the submitted version.

## Conflict of Interest

The authors declare that the research was conducted in the absence of any commercial or financial relationships that could be construed as a potential conflict of interest.
